# Metabolic decline in an insect ear: correlative or causative for age-related auditory decline?

**DOI:** 10.3389/fcell.2023.1138392

**Published:** 2023-05-18

**Authors:** Thomas T. Austin, Christian L. Thomas, Clifton Lewis, Alix Blockley, Ben Warren

**Affiliations:** Neurogenetics, College of Life Sciences, University of Leicester, Leicester, United kingdom

**Keywords:** audition (physiology), sensory decline, metabolism, aging, insect hearing

## Abstract

One leading hypothesis for why we lose our hearing as we age is a decrease in ear metabolism. However, direct measurements of metabolism across a lifespan in any auditory system are lacking. Even if metabolism does decrease with age, a question remains: is a metabolic decrease a cause of age-related auditory decline or simply correlative? We use an insect, the desert locust *Schistocerca gregaria*, as a physiologically versatile model to understand how cellular metabolism correlates with age and impacts on age-related auditory decline. We found that auditory organ metabolism decreases with age as measured fluorometrically. Next, we measured the individual auditory organ’s metabolic rate and its sound-evoked nerve activity and found no correlation. We found no age-related change in auditory nerve activity, using hook electrode recordings, and in the electrophysiological properties of auditory neurons, using patch-clamp electrophysiology, but transduction channel activity decreased. To further test for a causative role of the metabolic rate in auditory decline, we manipulated metabolism of the auditory organ through diet and cold-rearing but found no difference in sound-evoked nerve activity. We found that although metabolism correlates with age-related auditory decline, it is not causative. Finally, we performed RNA-Seq on the auditory organs of young and old locusts, and whilst we found enrichment for Gene Ontology terms associated with metabolism, we also found enrichment for a number of additional aging GO terms. We hypothesize that age-related hearing loss is dominated by accumulative damage in multiple cell types and multiple processes which outweighs its metabolic decline.

## Introduction

### Metabolic basis of age-related auditory decline—correlative or causative?

As we age, cellular metabolism declines and we succumb to age-related hearing loss. This has led to the hypothesis that metabolic decline is, in part, responsible for age-related hearing loss (Vaden et al., 2017; 2022). However, both aging and hearing loss have complex multicellular and multifaceted cellular bases. Causes of cellular aging include decreased proteostasis, epigenetic regulation, DNA damage, increased inflammation, mitochondrial dysfunction, and decreased cellular genesis (McHugh and Gil, 2018). Age-related hearing loss has been attributed to loss of auditory receptors (Coleman, 1976; Keithley and Feldman, 1982; Bhattacharyya and Dayal, 1985; Li and Hultcrantz, 1994), loss of auditory receptor synapses (Kujawa and Liverman, 2006; Kujawa and Liverman, 2009), degradation of the auditory nerve (Xing et al., 2012; Altschuler et al., 2015; Panganiban et al., 2022), and deterioration in the supporting cells that establish electrochemical gradients (Cable et al., 1993; Ohlemiller, 2009).

The three main causes of hearing loss were historically identified as loss of hair cells, loss of the spiral ganglion neurons (that form the auditory nerve), and deterioration of the stria vascularis in the lateral wall (Schuknecht and Gacek, 1993). Stria vascularis deterioration was thought to be the dominant cause of age-related hearing loss as it performs the metabolically demanding process of maintaining the endocochlear potential, which is responsible for efficient transduction and amplification. Recent analysis has spread doubt on this interpretation; some mouse models do not show a decrease of the endocochlear potential (Liu et al., 2022). Also, although stria vascularis atrophy is the most consistent finding (Schuknecht and Gacek, 1993) in older cochleae, the loss of hair cells best predicts the hearing function in humans (Wu et al., 2020). Going a step further, researchers over the last two years have analyzed changes in auditory receptors that survive; thus, going beyond, cell counting alone as a way to predict age-related loss of auditory function (Blockley et al., 2002; Keder et al., 2020; Jeng et al., 2021).

Four out of seven age-related hearing loss-susceptible mouse models have an age-dependent decrease in the endocochlear potential (Ohlemiller, 2009). Separate from the lateral wall, the organ of Corti houses the auditory receptors. Optical measurements within the organ of Corti for the key metabolic enzyme nicotinamide adenine dinucleotide (NAD) show a reduction of its expression (Okur et al., 2022) and function (Majumder et al., 2019). Reduction of NAD gives a good indication of oxidative phosphorylation as one molecule of NADH is oxidized to NAD for every 3 ATP produced in the electron transport chain (Cooper, 2000). There is a clear precedent for a reduction in metabolism causing age-related hearing loss in mammals (Schmiedt et al., 2002). However, a lack of direct measurement of metabolism in auditory tissues and a lack of age-dependent correlation in natural aging models mean that this is far from proven.

**FIGURE 1:**
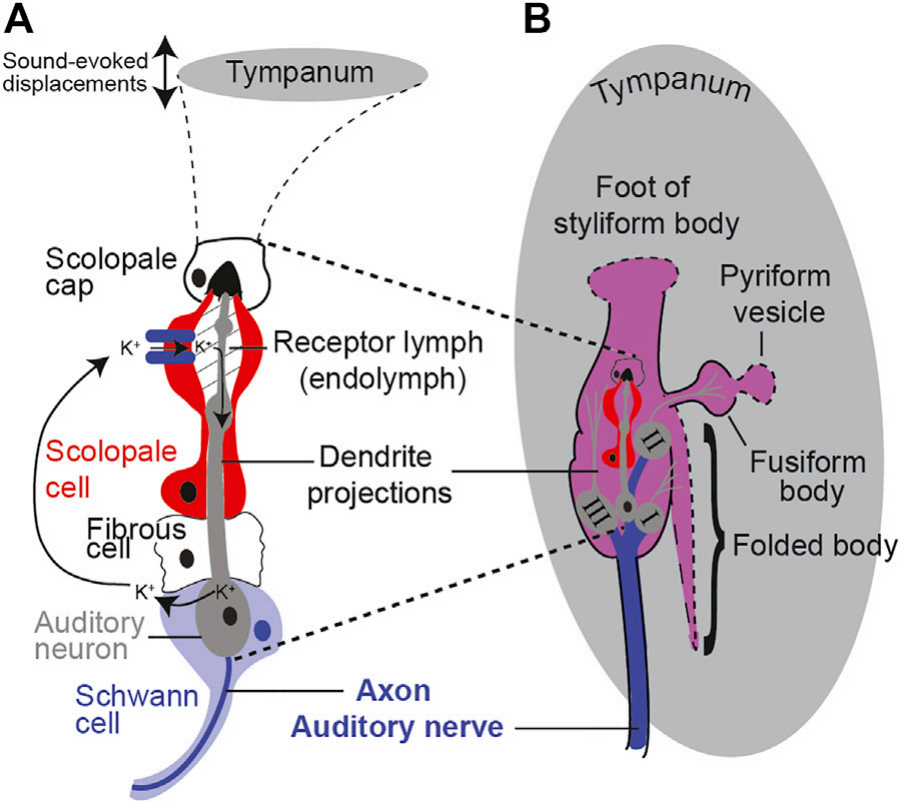
Schematic showing the anatomy and morphology of Müller’s organ (not to scale). **(A)** Morphology of a scolopidium including the auditory neuron, cap cell, ion-pumping scolopale cell, and cycle of cations. **(B)** Anatomy of Müller’s organ with three groups of auditory receptor neurons and their connections (dotted lines) onto the tympanum.

All auditory organs, from mammalian to invertebrate, share metabolically demanding physiological principles of function. These include specialized ion-pumping cells maintaining a potential across receptor cells and a spiking auditory nerve to carry auditory information to the central nervous system (Figure 1). Metabolism is as ancient as life itself and evolved over 3 billion years ago, 2.8 billion years before the evolutionary split between invertebrates and vertebrates. As well as sharing the metabolic demands of hearing, like all other ear-possessing animals, the desert locust manifests an age-dependent auditory decline (Blockley et al., 2002; Keder et al., 2020). Age-related auditory decline in the locust is measured as a decrease in the sound-evoked auditory nerve activity and a decrease in the number of auditory neurons (Blockley et al., 2002). Insects are emerging as a model to understand the basic principles of hearing loss including the genetic orchestration (Keder et al., 2020), molecular homeostasis (Boyd-Gibbins et al., 2021), biomechanics (Keder et al., 2020), and physiology (Blockley et al., 2002; Warren et al., 2020). Some of the advantages that insect models offer compared to established mammalian models are their efficient genetic toolkit (Keder et al., 2020), large number of experimental animals to detect subtle changes, physiological amenability (Blockley et al., 2002), short lifespan, low cost of maintenance, and fewer ethical barriers. On these bases, we justify using a high-throughput and physiologically malleable insect model, the tympanal ear of the desert locust, with its attached Müller’s organ (Figure 1).

Müller’s organ bridges across the inside surface of the tympanum and is composed of ∼80 auditory neurons of three different groups that can detect frequencies from 200 Hz to 40,000 Hz. Here, we focus on the function of Group III auditory neurons as these compose the majority of auditory neurons of Müller’s organ (∼46 out of ∼80) (Figure 1) (Jacobs et al., 1999). Group III auditory neurons are the most sensitive auditory neurons of Müller’s organ (Römer, 1976) and are broadly tuned to the 3 kHz pure tone we used for acoustic stimulation (Warren and Matheson, 2018). With the locust’s high-throughput approach, we measured the metabolic rate of the locust’s Müller’s organ. In addition, the locust’s physiological malleability enabled manipulations of ear metabolism to test if the metabolic state of the ear determines the auditory function. We predict that maintenance of the auditory neurons’ spike readiness and maintenance of the receptor lymph cavity’s electrochemical potential will be the most metabolically demanding processes in Müller’s organ. Our working hypothesis is to test if the presumed decrease in metabolism is causative for age-related auditory decline. If metabolic manipulations of Müller’s organs fail to alter its function, this suggests that auditory decline is not caused by a decrease in metabolism.

## Results

### Age-dependent decrease in Müller’s organ metabolism

**FIGURE 2:**
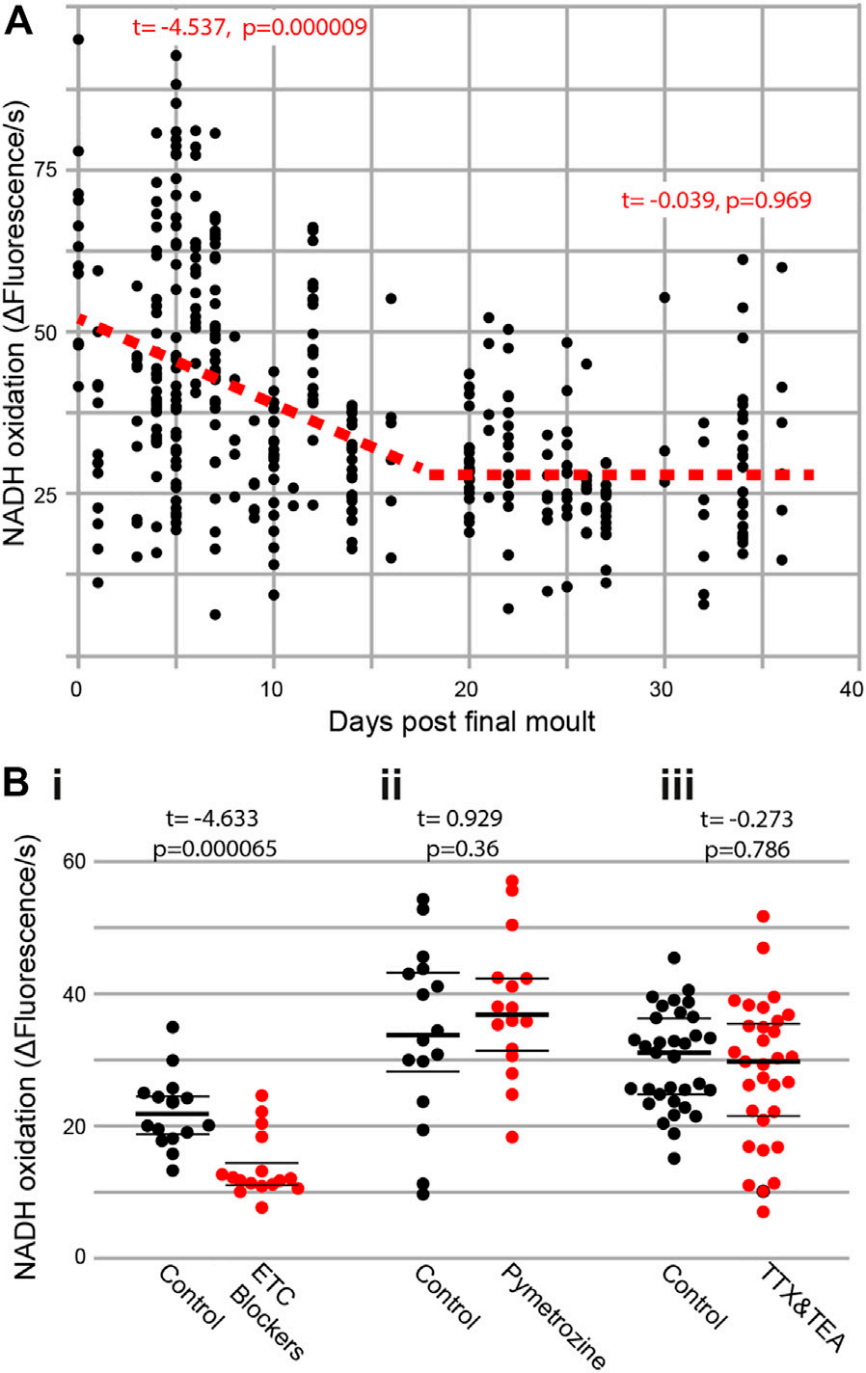
**(A)** Metabolic rate decreases with age and is not affected by interfering with auditory neurons. Rate of reduction of resazurin through NADH oxidation was measured fluorometrically in two ears from individual locusts as a function of their age post last moult [Cohen’s d = 0.902 (comparing days 0–5 with days 30–35)]. **(Bi)** The addition of electron transport chain blockers (Cohen’s d = 1.637), **(Bii)** auditory neuron-specific insecticide pymetrozine, and **(Biii)** sodium- and potassium-voltage gated ion channel blockers (TTX and TEA) on the rate of resazurin reduction.

To test if metabolism decreases in an age-dependent manner, we extracted Müller’s organs from locusts of known ages following their final moult. There was a clear decline in the rate of metabolism across all ages (Figure 2A) (t_(269)_ = −9.2, p < 1 × 10^−16^). However, if we split the data into two halves, of the lifespan measured, we find a decline between 0 and 16 days post final moult (t_(145)_ = −4.54, *p* = 0.000009) but no decline in metabolism between the ages of 20 and 34 days following final moult (t_(107)_ = −0.039, *p* = 0.969). Thus, the rate of metabolic decline decreases and plateaus with an increase in age. We used three blockers of the electron transport chain, sodium azide (1 mM), antimycin A (0.5 μM), and rotenone (0.5 μM), as positive controls for our metabolic assay and found a decrease in metabolic output (Figure 2Bi, t_(30)_ = −4.633, *p* = 0.0000656). To assess the metabolic involvement of action potential generation and auditory neuron function, we used tetrodotoxin (TTX) and tetraethylammonium (TEA) to block voltage-gated sodium and potassium channels and pymetrozine to silence auditory neurons. Both pharmacological interventions resulted in no change in the rate of metabolism (Figure 2Bii, TTX and TEA: t_(63)_ = -0.273, *p* = 0.786 and Figure 2Biii, pymetrozine: t_(30)_ = 0.929, *p* = 0.36).

### Unchanged spontaneous auditory nerve activity with age

**FIGURE 3:**
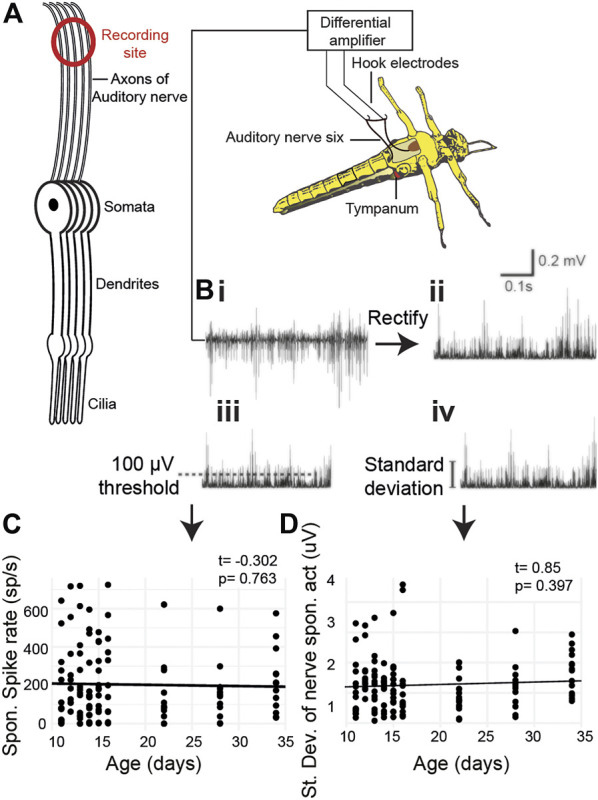
Metabolic activity of Müller’s organ measured as output from the auditory nerve. **(A)** Experimental setup of hook electrode recordings. **(Bi)** Spontaneous potentials from the auditory nerve, **(Bii)** rectified potentials from the auditory nerve, **(Biii)** 100-μV threshold used to count spontaneous spikes, and **(Biv)** standard deviation of the rectified nerve potentials. **(C)** Quantification of the number of spikes as a function of age. **(D)** Quantification of the standard deviation of the rectified nerve potential as a function of age.

We quantified age-related changes in spontaneous activity of Müller’s organ by recording electrical potentials from the auditory nerve using hook electrodes (Figures 3A,B). We recorded spontaneous auditory nerve activity from locusts 10 days to 34 days post their final moult (Figures 3A, Bi). We either used a threshold of 100 μV to count spikes (Figure 3Biii) or calculated the standard deviation of the auditory nerve spontaneous electrical activity (Figure 3Biv). The spontaneous spike rate did not change as a function of age (Figure 3C, t_(127)_ = 0.85, *p* = 0.397). The total electrical activity also did not change as a function of age (Figure 3D, t_(119)_ = -0.302, *p* = 0.763).

### Electrophysiological properties of auditory neurons

**FIGURE 4:**
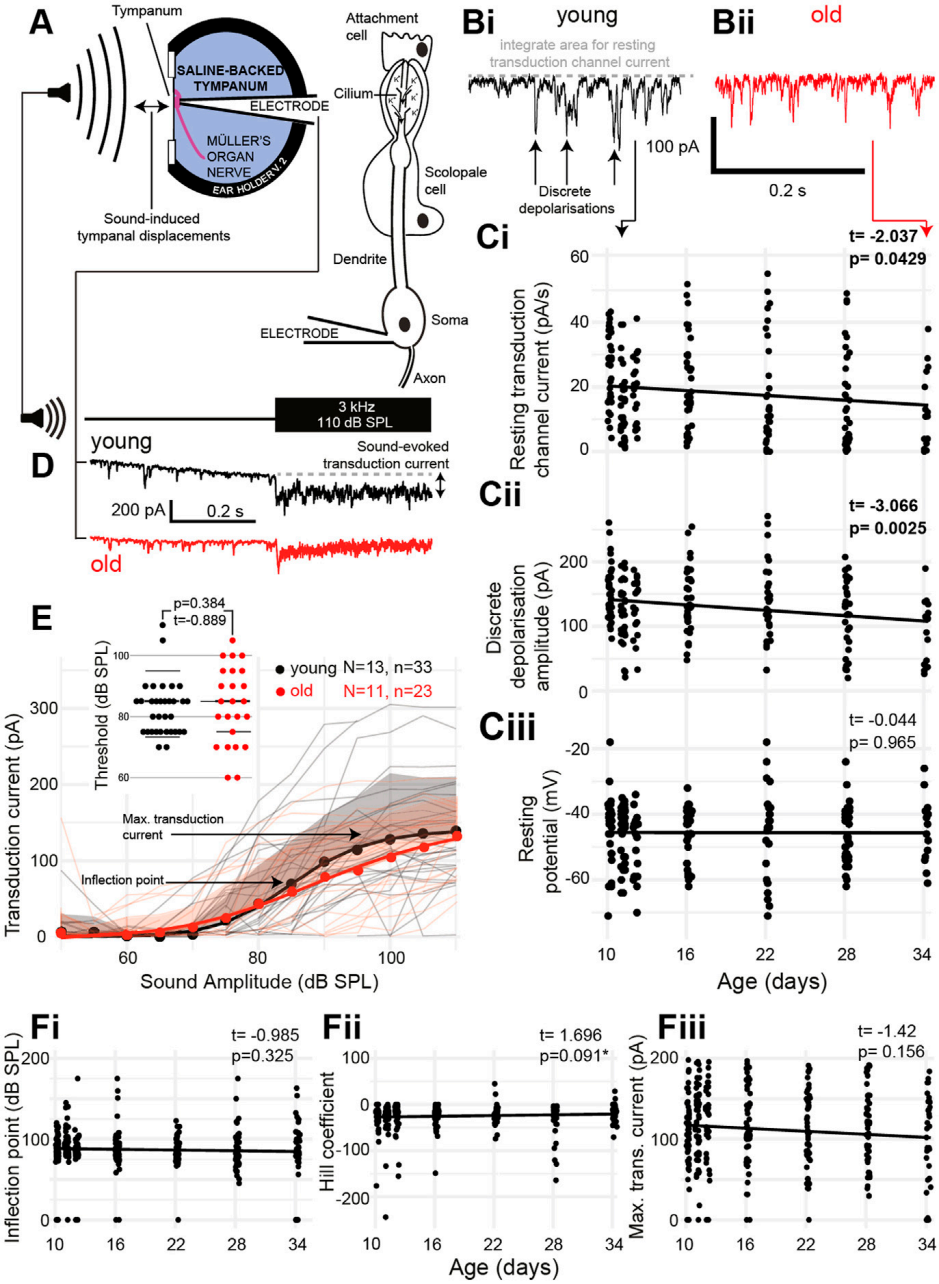
Properties of aging auditory neurons. **(A)** Experimental setup and recording configuration. **(B)** Voltage-clamp trace of auditory neuron resting transduction channel stochastic openings (discrete depolarizations) from **(Bi)** young (black) and **(Bii)** old (red) (34 days post final moult) locust ears. **(Ci)** Quantification of the amount of resting current flowing through the transduction channels as a function of age [Cohen’s d (comparing day 10 with day 34) = 0.837]. **(Cii)** Quantification of the amplitude of discrete depolarizations as a function of age [Cohen’s d (comparing day 10 and day 34) = 0.758]. **(Ciii)** Resting potential of the auditory neurons as a function of age. **(D)** Example of sound-evoked depolarization measured in the voltage-clamp mode for young (black) and old (red) locusts. **(E)** Quantification of the transduction current as a function of sound amplitude for young (black) and old (red) locusts. Dots are average, solid lines are a log-linear four compartment fit to the data, and the shaded area is the positive standard deviation for control (grey) and aged (pink) locusts. Thin lines are the measurements from individual locusts for young (grey) and old (pink) locusts. Insets are thresholds of a detectable transduction current for individual neurons in young and old Müller’s organs. **(Fi)** Inflection point (as shown in E) of the log-linear fits to transduction currents from individual locusts across ages. **(Fii)** Hill coefficient (shown in E) of the log-linear fits to transduction currents from individual locusts across ages **(Fiii)** Maximum transduction current (shown in E) of the log-linear fits to transduction currents from individual locusts across ages.

The auditory neurons of insects pass cations through their transduction channels, from the receptor lymph cavity, an enclosure of a high electrochemical gradient (analogous to the scala media endolymph in the cochlea). We pharmacologically blocked the spiking activity of the auditory neurons to only measure the activity of the transduction channels (Figure 4A). The transduction channels of auditory neurons spontaneously open even at rest to produce discrete depolarizations (Figures 4Bi, Bii). Both the amount of current that passed through the transduction channels (Figure 4Ci) and the amplitude of the discrete depolarizations (Figure 4Cii) decreased as a function of age (t_(200)_ = -2.037, *p* = 0.0429 and t_(201)_ = -3.066, *p* = 0.0025). The resting potential of the auditory neurons remained unchanged as a function of age [Figure 4Ciii (t_(195)_ = -0.044, *p* = 0.965)]. Next, we stimulated with 3-kHz tone across a range of sound amplitudes and recorded the transduction current whilst clamping the auditory neuron at −100 mV to increase the driving force of cations into the neuron (Figure 4D). We measured the transduction current because it allowed us to infer the electrochemical gradient of the receptor lymph cavity. We predict that maintenance of the receptor lymph electrochemical gradient is a metabolically demanding process. We fitted a four-part log-linear function to the transduction current as a function of the sound amplitude for each recording (Figure 4E, log-linear for the average data). We also plotted the threshold sound amplitude for each auditory neuron but found no difference (Figure 4E, inset). The inflection point was not different between young and old locusts’ auditory neurons (Figure 4Fi) (t_(203)_ = 0.985, *p* = 0.325) and neither was the Hill coefficient (Figure 4Fii) (t_(203)_ = 1.696, *p* = 0.091). The maximum transduction current produced with the largest sound amplitude (110 dB SPL) allowed a measure of the electrochemical potential of the receptor lymph cavity (Warren et al., 2020). The maximum transduction current did not significantly decrease as a function of age (Figure 4Fiii) (t_(230)_ = −1.42, *p* = 0.156).

### Individual differences in metabolism and sound-evoked auditory nerve activity in the same Müller’s organ

**FIGURE 5:**
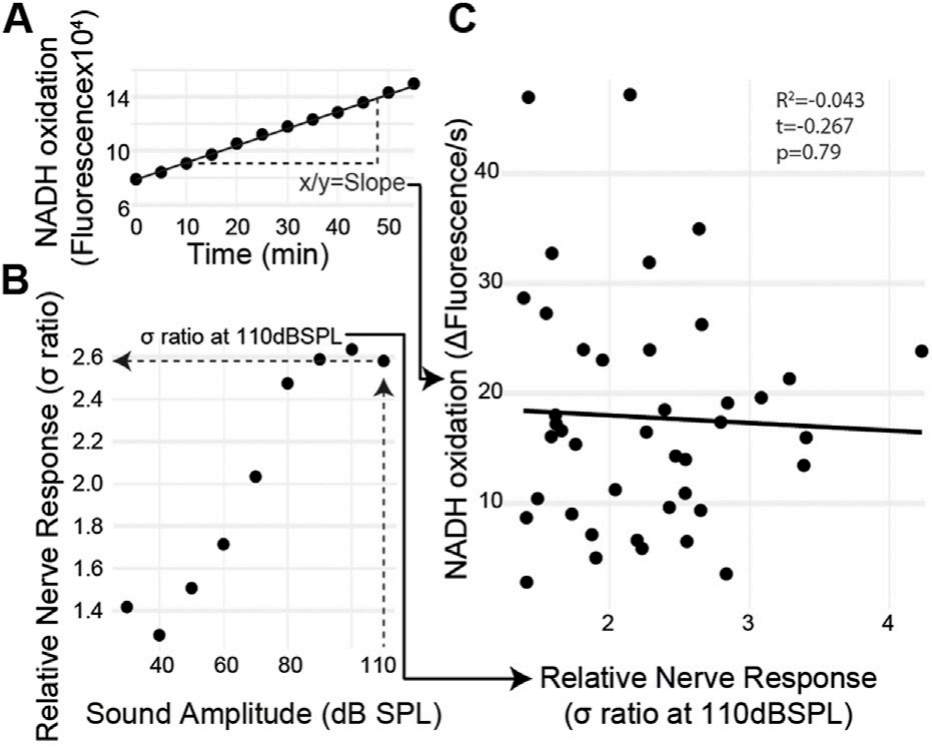
Ear-specific correlation of the sound-evoked nerve activity and metabolism. **(A)** Metabolic rate was measured by quantifying the slope of fluorescent changes as a function of time. **(B)** Maximum nerve response, the *σ* ratio, was measured at 110 dB SPL. **(C)** Both measurements were plotted for each ear with no correlation between the auditory nerve function and metabolic rate.

If metabolism is causative for age-related auditory decline, then auditory organs with a lower metabolic rate should have decreased auditory function regardless of age and *vice versa*. The metabolic rate and the sound-evoked auditory nerve response varied among individuals. Therefore, we measured sound-evoked nerve activity in an individual ear and then extracted the same ear and measured its metabolic rate using the resazurin metabolic assay (Figure 5A). The ear’s metabolic rate was quantified within 10 min of finishing the hook electrode recording (Figure 5B). We found no correlation between the auditory nerve response and the rate of metabolism (Figure 4C) (t_(39)_ = 0.267, *p* = 0.79) even with a large variation in both measurements.

### Cold-rearing locusts increased Müller’s organ metabolism

**FIGURE 6:**
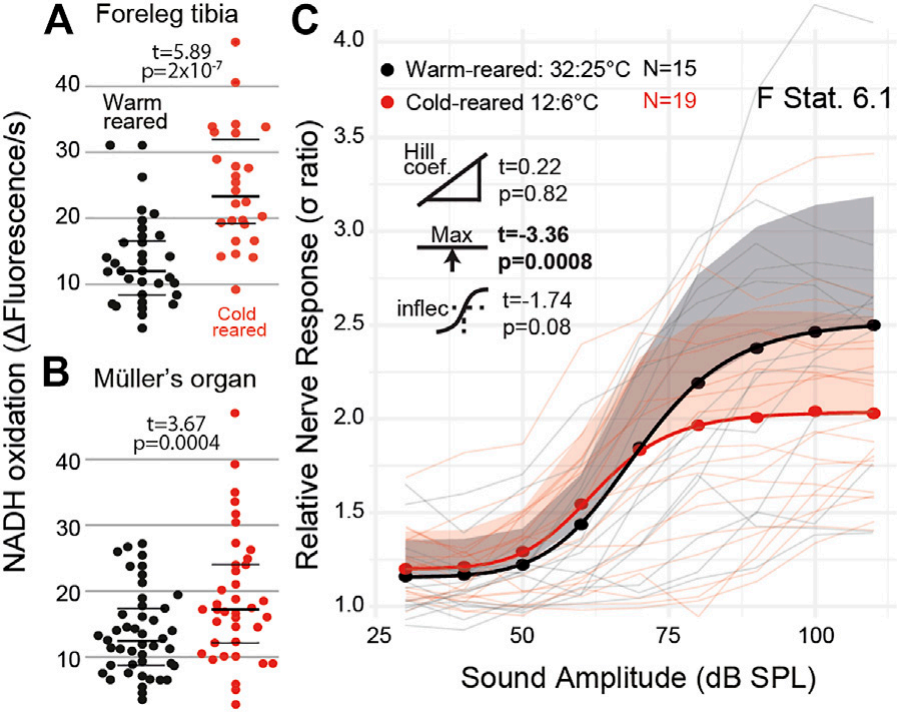
Quantification of the metabolic rate and auditory nerve function in cold-reared locusts. **(A)** Metabolic rate of reduction of resazurin through NADH oxidation increased in the foreleg tibia under normal (32:25°C day: night) conditions and cold (12:6°C day:night) conditions (Cohen’s d = −2.08). **(B)** Metabolic rate of Müller’s organs in warm- and cold-reared locusts (Cohen’s d = −0.57). **(C)** Sound-evoked responses of locusts reared under warm and cold conditions with respect to sound amplitude. Responses from individual locusts are plotted as thin lines (pink = cold-reared; black = warm-reared), and averages for warm- and cold-reared locusts are dots. The positive standard deviation of the averages are shaded regions, and the solid thick lines are the four-part log-linear fit to the average responses (Cohen’s d = 0.793 (model fit at 110 dB SPL)).

We exploited the ectothermic nature of the locust to manipulate chemical reaction rates through altering the environmental temperature. We raised locusts either under standard environmental conditions (32:25°C day:night) or cold conditions (12:6°C day:night). We successfully altered the metabolic rate of both muscle tissues in the foreleg tibia (Figure 6A, t_(27)_ = 5.89, p = 2 × 10^−7^) and Müller’s organ (Figure 6B, t_(43)_ = 3.67 *p* = 0.0004). The sound-evoked response of the auditory nerves was different among locusts raised in cold conditions (Figures 6C: F Statistic = 6.12). This difference was manifested by a lower maximum asymptote of the auditory nerve response in cold-reared locusts, opposite to the increase of the metabolic rate in Müller’s organs (t_(4)_ = -3.37, *p* = 0.00086). The other three parameters of the four-part log-linear fit were not significantly different (Hill coefficient t_(4)_ = 0.22, *p* = 0.82; lower asymptote t_(4)_ = 0.20, *p* = 0.84; and inflection point t_(4)_ = -1.74, *p* = 0.08).

### Starving locusts reduced Müller’s organ metabolism

**FIGURE 7:**
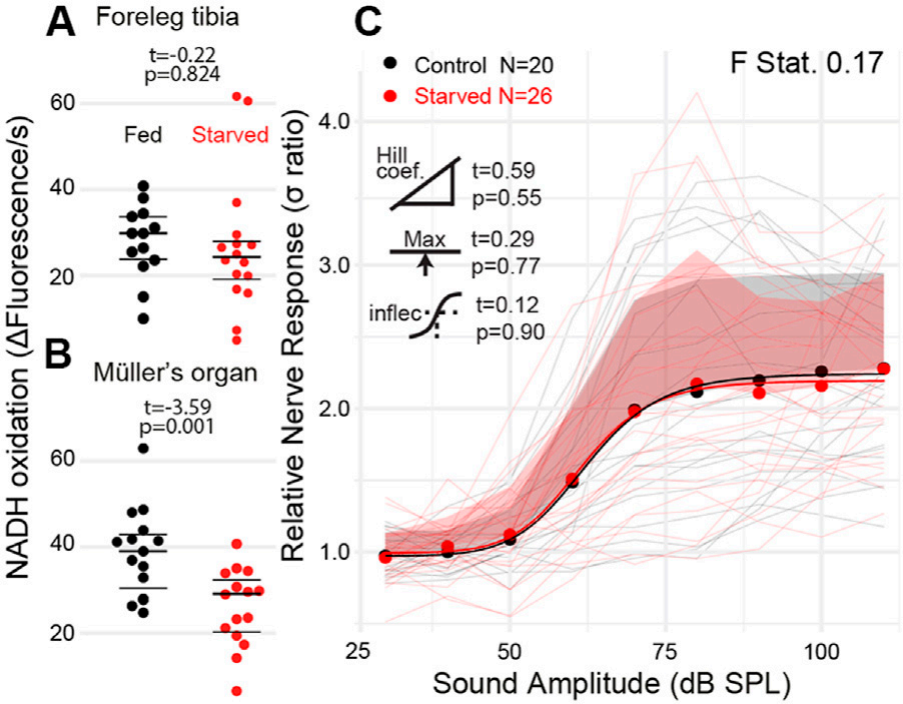
Quantification of the metabolic rate and auditory nerve function in starved locusts. **(A)** Metabolic rate of reduction of resazurin through NADH oxidation in the foreleg tibia in locusts fed *ab libitum* and starved locusts. **(B)** Metabolic rate of Müller’s organs in locusts fed *ab libitum* and starved locusts (Cohen’s d = 1.31). **(C)** Sound-evoked responses of locusts fed *ab libitum* and starved locusts. Responses from individual locusts are plotted as thin lines (pink = starved, black = fed), and averages for starved and fed locusts are dots. The positive standard deviation of the averages is the shaded regions, and the solid thick lines are the four-part log-linear fit to the individual responses.

We starved 10-day old locusts for 6 days to attempt to alter the metabolic rate of the locust’s Müller’s organ independent of their age. We failed to reduce the metabolic rate of muscle tissue in the foreleg tibia (Figure 6A t_(27)_ = 0.22, *p* = 0.824); however, the metabolic rate of Müller’s organs was reduced compared with locusts fed *ab libitum* (Figure 7B t_28_ = −3.59, *p* = 0.001). The sound-evoked response of the auditory nerves across sound pressure levels was not significantly different between starved and fed locusts (Figures 7C: F statistic = 0.17) for any of the four parameters of the log-linear fit (maximum asymptote t_(4)_ = 0.29, *p* = 0.77; Hill coefficient t_(4)_ = 0.59, *p* = 0.56; lower asymptote t_(4)_ = 0.01, *p* = 0.99; and inflection point t_(4)_ = 0.12, *p* = 0.90).

### Gene expression changes with age

**FIGURE 8:**
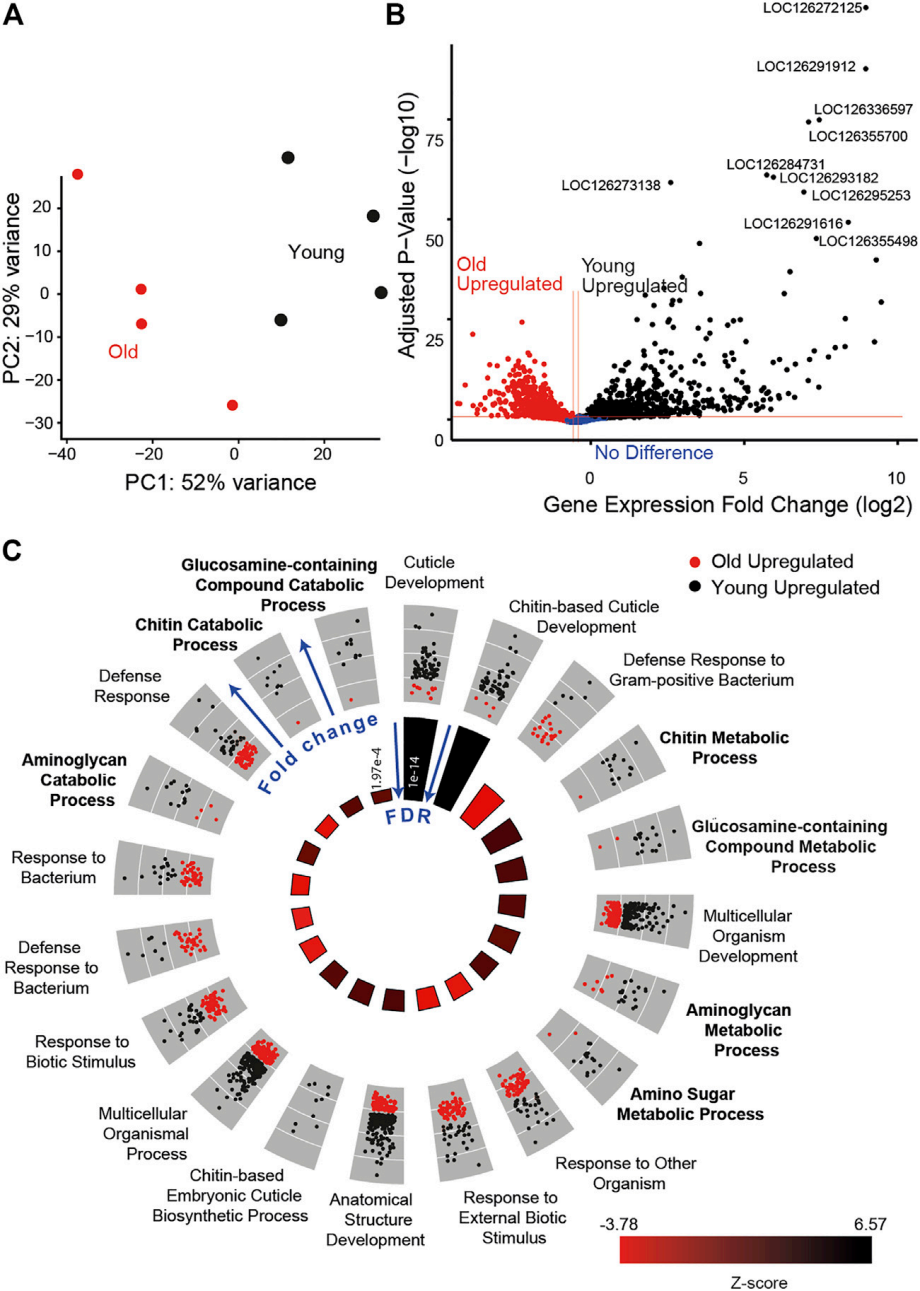
Transcriptomic results for young (black) and old locusts’ (red) Müller’s organs. **(A)** Principal component analysis of gene expression counts. **(B)** Volcano plot comparing the log-fold *p*-value and log-fold change, annotated with the top 10 differentially expressed gene’s LOC numbers. **(C)** GO circle plot of the top 20 enriched biological process GO terms. Each gene associated with the GO term is plotted in relation to its fold change, either being upregulated in old (red) or upregulated in young (black). In the inner circle, plots for the z-score are reported, which give an indication as to whether the group of genes associated with a particular GO term are more upregulated in young (black) or old (red) locust ears, with the size of the bar indicating the significance of the false discovery rate (FDR) (taller bars being more significant).

To further examine the role of metabolism in age-associated auditory decline, we compared the transcriptome of Müller’s organs between young and old locusts. We saw a clear separation between the two conditions using principal component analysis (Figure 8A). Transcriptomic results showed 1,109 differentially expressed genes between young and old locusts. A total of 486 genes were upregulated in the younger locusts, whilst 623 genes were upregulated in the older locusts (Figure 8B). The study by Keder et al. (2020) found 109 Johnston’s organ genes to be differentially expressed with age. We found 65% (71/109) of these differentially expressed JO genes to have orthologs in the locust genome. Of these genes, we only found two to be differentially expressed between young and old Müller’s organs. These were *Odf3* and *pyx*. After annotating the *Schistocerca gregaria* genome with Gene Ontology (GO) terms, we performed GO enrichment on the differentially expressed genes. We obtained 16 GO terms for cellular components, 20 for molecular functions, and 85 for biological processes.

Many of the most enriched biological GO terms are for terms associated with chitin development, such as cuticle development, chitin-based cuticle development, and the chitin metabolic process. This is due to the large number of differentially expressed genes associated with the chitin-based process. For example, among the top 20 most significant genes are cuticle protein 65 (LOC126291912 and LOC126355498), cuticle protein 16.5 (LOC126291616), and cuticle protein 5 (LOC126355435). We suspect the high number of differentially expressed genes associated with chitin development is a consequence of the attached tympanum, as in the desert locusts, chitin production decreases significantly as a function of age following final moult (Candy & Kilby, 1962).

Although the immune system process was not enriched in this analysis, a number of GO terms associated with immunity were found to be significantly enriched, including defense response to the Gram-positive bacterium, response to external biotic stimulus, and activation of innate immune response (Figure 8C). The genes associated with these GO terms were generally upregulated in the old locusts. In addition, among the top 100 differentially expressed genes were genes including hemocytin (LOC126356178) and two *Mucin-19* genes (LOC126295440 and LOC126272492). In addition to *Mucin-19*, we find differentially expressed *Mucin-5AC* (*MUC5ac*) (LOC126281354). *MUC5ac* overexpression has been associated with hearing loss (Lee et al., 2015), although paradoxically it was upregulated in the young locust. We also found enrichment for the inflammatory response GO term, with genes such as *Mucin-17* (LOC126337069), nuclear factor NF-kappa B p110 subunit (LOC126282030), and peptidoglycan recognition protein 1 (LOC126267215 and LOC126267215).

Other interesting enriched biological process GO terms included cytolysis and response to estrogen. Additionally, we identified some genes which had previously been implicated in the hearing loss. Some of these genes include *RXR* (LOC126272034), *eEF2* (LOC126272205), and *SLC52A3* (LOC126272245).

Focusing primarily on metabolism, we found enriched GO terms associated with metabolic processes including the aminoglycan metabolic process, amino sugar catabolic process, and carbohydrate derivative catabolic process (Figure 8C). A total of 274 metabolic process-associated genes were found to be differentially expressed. We found some NAD metabolism-associated genes to be differentially expressed; L-lactate dehydrogenase (*LDH*) (LOC126266619) and pyruvate kinase (LOC126274964) both show increased expression with age, and glycerol-3-phosphate dehydrogenase (LOC126266753) shows decreased expression with age.

## Discussion

Metabolism is hypothesized to be responsible for the age-related hearing loss. However, the metabolic rate has never been measured as a function of age in an auditory organ and neither has metabolism been manipulated to establish a causative role. Using an insect model, we measured the metabolic rate in an auditory organ by quantifying the reduction of resazurin, by nicotinamide adenine dinucleotide (NAD), a redox signaler and key co-enzyme for glycolysis and oxidative phosphorylation. We then manipulated the metabolic rate in an attempt to find a causative role of metabolism in age-related auditory decline. Finally, we measured gene expression changes as a function of age in the locust’s specialized auditory organ, Müller’s organ.

We found that the metabolic rate is lower in Müller’s organs in older locusts paralleling that found across whole-body basal metabolism in a range of animals (***Drosophila*:** Fiorino et al., 2018; **Guppies**: Imai et al., 2021; **Mice**: Haramizu et al., 2011; and **Humans**: Henry, 2005). The slowing of metabolism later in life is also paralleled by basal metabolic measurements in humans (Henry, 2005). Whilst it is not surprising that metabolism decreased in Müller’s organs with age, it could also be hypothesized that auditory tissue evolved a special resilience against metabolic-dependent age-associated deterioration due to the selective advantage of sensitive hearing for predator avoidance and mate finding.

We predicted that interfering with metabolically demanding processes of the auditory neurons through the application of voltage-gated ion channel blockers and a chordotonal organ-specific insecticide, pymetrozine, would result in a decrease in metabolism, as the neurons would not be able to spike. We would expect pymetrozine to have a severe effect on metabolism as it chronically opens the heteromeric TRP channel Nanchung-inactive, leading to depolarization in the auditory neuron and a decrease in the receptor lymph potential. In the neuron, pymetrozine initially leads to spiking (Warren and Matheson, 2018), but later, a silent non-spiking neuron presumably requires less energy to maintain spike readiness. However, the decrease in the receptor lymph potential could result in the supporting scolopale cell increasing its metabolic rate to maintain the receptor lymph potential through fuelling transmembrane ion exchangers. Thus, compensatory mechanisms could mask localized changes when we measured the metabolic rate of the entire Müller’s organ and attached the auditory nerve. When blocking voltage-gated sodium and potassium channels and, thus, spiking (with TTX and TEA), we would expect a decrease in the neurons’ metabolism. We found no change in Müller’s organ metabolism upon application of TTX and TEA or pymetrozine. It is likely, in Müller’s organ, that the combined metabolic demands of the primary auditory receptors do not outweigh those of the more numerous supporting cells. The primary auditory receptors comprise 5% of cells in the auditory organ (∼80 auditory neurons out of 1,500 total cells in Locusts) (Blockley et al., 2002). Much of the cellular energy budget is required to maintain protein homeostasis within a cell. For example, even in the human brain, 25% -50% of the energy consumption is not related to signaling (Engl and Atwell, 2015), despite the brain having a much higher proportion of neurons compared to Müller’s organ.

Although the number of auditory neurons decreases as a function of age (Blockley et al., 2002), we found that there is no age-dependence in the number of spontaneous spikes travelling along the auditory nerve (Figure 3C). At the level of the auditory receptors, we found a decrease in the passing current through the transduction channels, which mirrors that found in mouse hair cells (Jeng et al., 2021). We inferred the receptor lymph potential from the magnitude of the transduction current at maximal sound amplitude (Warren and Matheson, 2018). We assume no change in the number or biophysical properties of the transduction channels. The expression of the transduction ion channel candidate genes, *NompC*, *Nanchung*, and *Inactive*, remains unchanged between young and old locusts. The unchanged age-dependent expression on *Nanchung* conflicts with changes in the *Drosophila* auditory organ, Johnston’s organ (Keder et al., 2020). We cannot rule out changes in the transduction channels’ individual biophysical properties as has been shown in the fruit fly Johnston’s organ (Keder et al., 2020) but such changes are compensatory. We found no significant (t = −1.42) age-related decrease in the maximal sound-evoked transduction current reflecting the receptor lymph potential. We believe that the reduction of the passing current through the transduction channels could be a common mechanism to maintain the electrochemical potential of the receptor lymph to compensate for an age-dependent decrease in the performance of ion-pumping scolopale cells or, in the case of mammals, cells of the lateral wall in the cochlea. Although we did not measure a significant decrease in the receptor lymph potential, we still conclude that there was an age-dependent decrease in the ability of the scolopale cell to maintain the lymph’s high gradient for the following reason: if the number of cations pumped into the receptor lymph remains unchanged through aging and if there is less leakage of cations from the receptor lymph into the auditory neuron (standing transduction current), then we would expect a higher receptor lymph potential. As we find no change, if anything, a mild decrease in the inferred receptor lymph potential (t = −1.42), we must conclude that the scolopale cells are pumping fewer ions into the receptor lymph space as they age. The resting membrane potential of the auditory neurons is well maintained into old age, similar to mouse hair cells (Jeng et al., 2021). Altogether, the metabolic demands of the auditory neurons appear to remain fairly constant over its lifespan, with a possible mechanism to compensate for age-related decline in the performance of ion-pumping cells.

We have limited our scope to observing changes in proton availability, produced in oxidative reactions throughout Müller’s organ. A large proportion of these protons will come from oxidative phosphorylation, where NADH is oxidized to NAD+, which the resazurin assay predominantly measures. Metabolic homeostasis is complex, with regulatory and compensatory mechanisms existing both within individual cells and compensating for other cell types within a system. For instance, individual cells also use different pathways and metabolites available under different forms of metabolic stress, re-wiring fundamental metabolic pathways (Vincent et al., 2015). Within a multicellular nervous system, Schwann cells transfer metabolic substrates to axons (Vega et al., 2003) to regulate metabolic homeostasis within neurons. This highlights the diverse metabolic specialisms within a system; neurons are mostly oxidative metabolizers, whereas supporting (glial) cells are predominantly glycolytic (Fünfschilling et al., 2012). It is possible that auditory neurons may be buffered against fluctuations in metabolic resource supply and demand by supporting cells.

Here, we wished to determine if whole auditory organ metabolism directly determined its performance, or if metabolic performance is simply a correlative proxy for age. Decreasing metabolism through dietary restriction is shown to extend lifespan in diverse organisms (McCay et al., 1935; Klass, 1977; Partridge et al., 1987; Anderson et al., 2003) presumably due to a decrease of oxidative stress, enhanced mitochondrial efficiency (Ruetenik & Barrientos, 2015), decreased DNA damage (Haley-Zitlin & Richardson, 1993), and a decreased immune response (Wu, 2019). At the same time, a more metabolically active auditory organ would be expected to perform better, especially if this equates to an increased electrochemical gradient across the transduction channels (Schmiedt et al., 2002). Whilst we showed that dietary restriction altered the Müller’s organ metabolic rate (Figure 7), we observed no changes in the auditory nerve response to sound. Glucose deprivation (which we assume a locust experiences upon dietary restriction) causes cells to prioritize oxidative phosphorylation (Liu et al., 2003; Ahmad et al., 2005). Thus, our ability to measure the predominant form of metabolism (oxidative phosphorylation with the resazurin sodium assay) would improve with dietary restriction. In addition, we successfully manipulated metabolism of locusts by rearing adults under colder conditions. However, we are surprised to find a corresponding decrease in the cold-reared locusts’ auditory response relative to their warm-reared counterparts (Figure 6).

Given that our manipulations of the metabolic rate failed to alter the auditory response, we investigated age-related gene expression changes in Müller’s organs to identify additional mechanisms responsible for the age-related hearing loss. We find GO term enrichment for some metabolic processes, such as the aminoglycan metabolic process, amino sugar catabolic process, and carbohydrate derivative catabolic process, but we also found enrichment for a number of other aging processes. One aging hallmark, chronic inflammation, is contributed by oxidative stress, age-related changes in the inflammatory cytokine network, and cellular senescence (Kim & Song, 2014). Immune response genes were clearly upregulated in older Müller’s organs paralleling that found in the mouse cochlea (Schubert et al., 2022). We also found genes enriched for cytolysis to be differentially expressed between young and old locust auditory organs. These findings add further evidence to the multi-process nature of age-related auditory decline. Similar to the study by Keder et al. (2020), GO terms associated with upregulated genes are associated with more homeostatic processes including immune response, metabolism, and amino acid homeostasis, whilst genes upregulated in younger locusts are more associated with chitinous processes.

Of the 109 differentially expressed auditory organ-specific genes found in *Drosophila* (Keder et al., 2020), we only found two to be differentially expressed between our young and old time points. This could be indicative of big differences in the age-related hearing loss between *Drosophila* and the locust, or could be a consequence of the larger number of days between young and old flies and locusts (Keder et al., 2020). However, we did find a number of differentially expressed genes that have been implicated with hearing loss in mammalian systems. For example, *RXR*, a nuclear receptor that decreases in expression with age, has been touted as a potential therapeutic target for the inner ear (Kwak et al., 2019). Here, we found *RXR*’s expression is reduced in older locust Müller’s organs compared with younger locusts. In mammals, the autophagy gene *eEF2*’s repression can alleviate many aging phenotypes and is found to be upregulated in severe hearing loss (Peng et al., 2022). Between young and old locusts, we found *eEF2* to be upregulated in the older locusts. A final example is *LDH*. *LDH* reversibly catalyzes the reaction of lactate to pyruvate. The *LDH* gene upregulated in aged locusts is most similar to *LDH-A*, as was also found in mice (Ross et al*.*, 2010). This increases the pyruvate-to-lactate reaction, which has previously been implicated in human age-related hearing loss (Tian et al., 2020). The enriched GO term for melanin biosynthesis also appears to increase with age, which may have a protective quality; melanin precursors have been shown to prevent both age-related and noise-induced hearing loss in mice (Murillo-Cuesta et al., 2010). In humans, black individuals also have a lower risk of hearing loss than their white counterparts, perhaps due to having increased inner ear melanin (Agrawal et al., 2008).

Given our inability to the alter auditory function following metabolic rate manipulations and the complexity of our transcriptomic results, our findings reopen the question of what is the key driver of age-related auditory decline. Studies until now have observed age-related changes in endocochlear potential (Schmiedt et al., 2002), and the field has attributed these to changes in the metabolic rate. Here, for the first time, we perform an auditory organ metabolic assay, and whilst we find the expected age-related metabolic decline, we fail to correlate the metabolic rate with the hearing ability. This echoes other hypotheses in the field (Wu et al., 2020; Liu et al., 2022) where hair cell loss, not stria vascularis atrophy, explain hearing loss. Our findings of the co-occurrence of a decline in the auditory function and metabolism could be explained by cumulative damage caused by generic aging processes (López-Otín et al., 2013) as opposed to solely the current metabolic rate of the locust’s Müller’s organ.

## Conclusion

We measured a decrease in the metabolic rate in the auditory organ of the desert locust as a function of lifespan. Auditory neurons showed little age-related changes in function and did not contribute significantly to the metabolic rate. We found no correlation between the metabolic output of individual Müller’s organs and the ability of the same Müller’s organ to transduce sound-evoked potentials. We altered the metabolic rate of the auditory Müller’s organ through dietary restriction and cold-rearing and found no change or opposite changes in the magnitude of its sound-evoked response. Age-related changes in gene expression suggest that although metabolic gene expression changes occur between young and old locusts, a range of additional aging processes are also candidates for causing age-related hearing loss.

## Methods

### Locust husbandry and conditioning

Desert locusts, *Schistocerca gregaria* (mixed sex), were reared under 150–250 crowded gregarious conditions in 60 cm^3^ cages in their fast-aging gregarious state, where they can live up to two months. They had a 12-h light/dark cycle at 32:25°C and were fed on a combination of fresh wheat and bran *ad libitum*. The gregarious state of the desert locust contrasts with their isolated solitarious state, where they live for up to 9 months. The founding progeny of the Leicester lab strain was solitary copulating adults collected at the Akjoujt station ∼250 km northeast from Nouakchott, Mauritania, in May 2015.

We performed a survival assay of our crowded gregarious locusts. We found that 21 days post-final moult into adults more than half of the locust population had died. Very few locusts continued to die over the next 12 days until we stopped counting.

**FIGURE 9:**
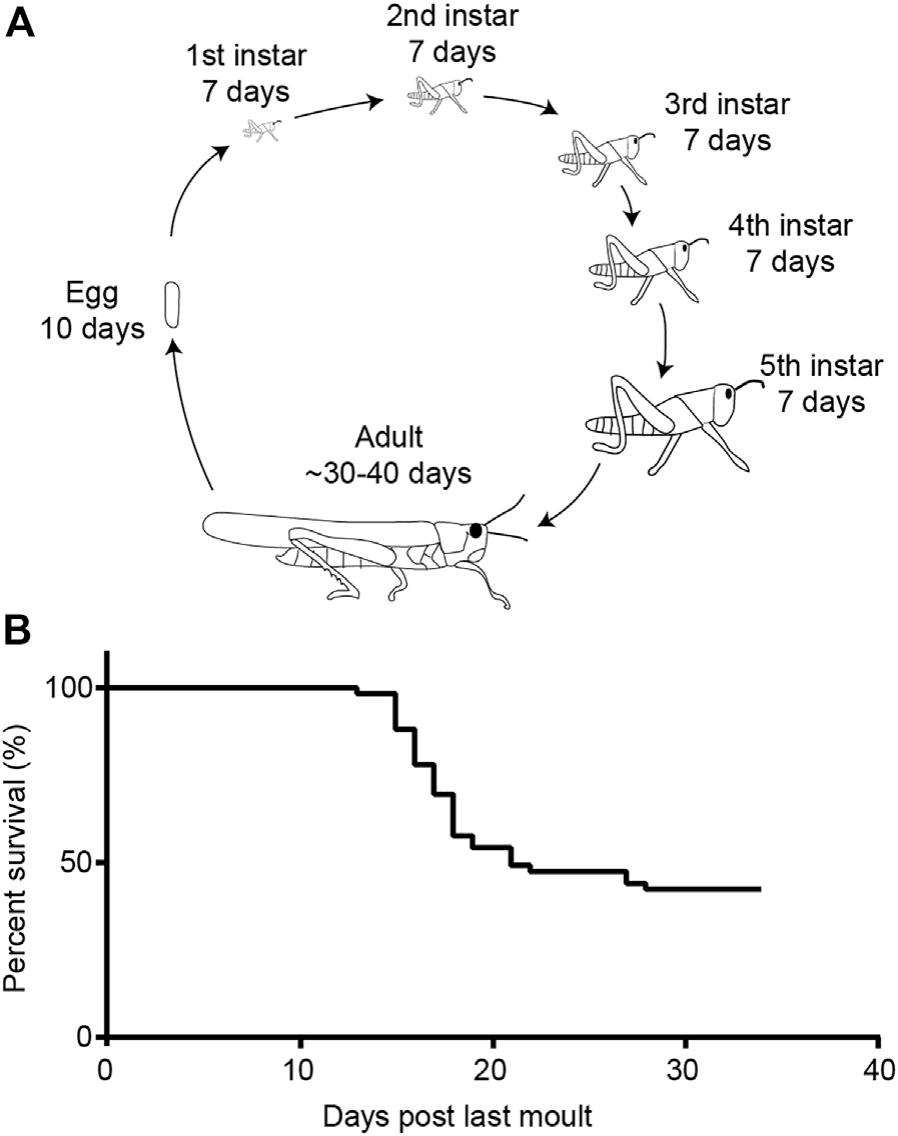
Life cycle of the desert locust and survival in the adult gregarious form. **(A)** Life cycle of the desert locust. **(B)** Life span of gregarious locusts under lab-reared conditions.

After hatching, desert locusts go through five moults before they become adults with functional wings. For the longitudinal metabolic assay (Figure 9), ∼20 locusts (mixed sex) were taken within 24 h of their final moult from their larger cages (previously described) and placed in plastic aquarium tubs (40 cm × 20 cm × 30 cm) with *ab libitum* wheat and milled bran.

For longitudinal hook electrode and patch-clamp recordings (Figures 3, 4), locusts (mixed sex) were taken 10 days post their last moult and had their wings clipped at their base. These locusts were aged in aquarium tubs as previously described.

The male locusts used to test the effect of TTX and TEA, electron transport chain blockers, and pymetrozine (Figures 2Bi–Biii) were all 10 days post their last moult. To test if the metabolic output of Müller’s organs is causative for auditory performance, and its decline, we used two approaches. In Figure 6, locusts 10 days past final moult were reared in a 12 h light/dark cycle at 12:6°C for 14 days. Control locusts were reared under standard conditions (32:25°C) as previously described for the same duration. Auditory nerve recordings and ear extractions were carried out at room temperature. In Figure 7, male locusts, 10 days past their final moult, were taken and raised under normal conditions, but without food for 7 days. Control animals were raised under normal conditions, fed *ad libitum*.

### Metabolic assay

A 0.02% (weight/volume) solution of resazurin sodium salt was dissolved in locust saline. Locust saline had the following concentrations (in mM): 185 NaCl, 10 KCl, 2 MgCl2, 2 CaCl2, 10 HEPES, 10 Trehalose, and 10 Glucose. The saline was adjusted to pH 7.2 using NaOH. Three other solutions were used as positive control, or to test the metabolic involvement of the auditory neurons. For the positive control, we used three electron transport chain blockers dissolved in resazurin sodium saline solution: 1 mM sodium azide, 0.5 μM antimycin A, and 0.5 μm rotenone. To selectively target the spiking activity of the auditory neurons, we used 90 nM TTX and 20 mM TEA, and to silence auditory neurons completely (see Warren and Matheson, 2018), we used 30 μM pymetrozine, all dissolved in resazurin sodium saline solution.

Volumes of 30 μL of the resazurin containing saline were pipetted into a 384 cell culture microplate with flat bottom and transparent lid (781,086, Greinerbio-one) on ice. Müller’s organs of locusts were extracted by inserting the Dumont #5 forceps (501,985, World Precision Instruments) so that each forcep point penetrated the tympanum (by approximately 0.2 mm) flanking the folded body. The forceps clasped Müller’s organ before pulling it away from the tympanum. The forceps were inspected to confirm extraction of Müller’s organ before repeating the process for the other ear of the locust. Both Müller’s organs were placed into a single well as shown in Figure 2A. We realized that this assay was sensitive for single ears, and as shown in Figures 2Bi–Biii, single Müller’s organs were placed in each well. To measure the metabolic rate of the tissue separate from Müller’s organ, we took the fore leg tibia and cut it on the tibia side of its joint. We placed it distal side up in the 384 Microwell plate well.

**FIGURE 10:**
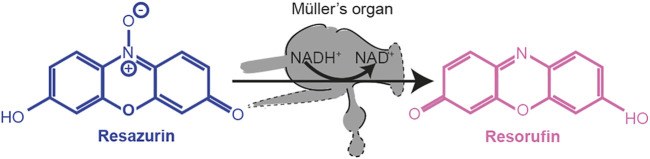
Reduction of resazurin to resorufin by using the metabolic co-enzyme NADH^+^ within Müller’s organ.

Resazurin (a weakly fluorescent blue dye) was oxidized by NADH into highly fluorescent resorufin (excitation/emission 571/584 nm) (Figure 10). Using the FLUOstar Omega BMG Microplate reader, we excited each well using an excitation filter at 544 nm and measured through an emission filter of 590 nm. Fluorescent intensity measurements with a gain of 1800 were taken every 5 min for 12 cycles. The increase in fluorescence was fitted with a regression line in Mars analysis software (BMG Labtech) to give an increase in fluorescent units per second.

### *In vivo* hook electrode recordings from auditory nerve six

Locusts were secured ventral side up with their thorax wedged in a plasticine channel and their legs splayed and held down with plasticine. A section of the second and third ventral thoracic segment was cut with a fine razor blade and removed with fine forceps. Tracheal air sacks were removed to expose nerve six and the metathoracic ganglia. This preparation left the abdomen, including the first segment where the ears reside intact, thus maintaining the operation of the ear *in vivo*. Hook electrodes constructed from silver wire 18 μm in diameter (AG549311, Advent Research Materials Ltd.) were hooked under the nerve, and the nerve was lifted out of the hemolymph. In Figure 3, a mixture of 70% Vaseline and 30% paraffin oil was applied through a syringe to coat the auditory nerve to stop it from drying out. In Figures 5–7, the nerve was not coated with a mixture of Vaseline and paraffin oil. Locust mounting and recordings took ∼15 min for each locust. In Figure 5, Müller’s organs were extracted as previously described directly after hook electrode recordings were completed (within 10 min). Signals were amplified 1,000 times by a differential amplifier (Neurolog System) then filtered with a 500-Hz high pass filter and a 50-kHz low-pass filter. This amplified and filtered data were sampled at 25 kHz by Spike2 (version 8) software running on Windows (version 10). To quantify the compound spiking activity of the auditory nerve, we used MATLAB (Version R2020a, Mathworks Inc.) and rectified the nerve signal and integrated the area underneath. We computed this for the 0.5 s of sound-evoked neural activity and for 60 s activity background nerve activity before the tones and the background activity between the tones. To compute the *σ* ratio, we rectified the nerve signal and removed any DC offset. We then divided the sound-evoked response by the background neural activity. In some batches of recordings, 3-kHz pickup was detected for the largest sound amplitude tones. These recordings were adjusted using a consistent multiplication factor to best remove the effect of this pickup from the data. For the Starved locusts (Figure 7), the locust treatment was blinded to the experimenter until all data were collected and analyzed. Blinding was not possible for the cold-reared experiments (Figure 6), as there was an observable phenotypic difference in cuticle colour and texture between the animals raised at different temperatures.

### Dissection of Müller’s organ and isolation of Group III auditory neurons

Whole-cell patch-clamp recordings were performed on Group III auditory neurons because they form the majority of auditory neurons of Müller’s organ (∼46 out of ∼80) (Figure 3A) (Jacobs et al., 1999), they are the most sensitive auditory neurons of Müller’s organ (Römer, 1976), and they are broadly tuned to the 3 kHz we used for noise exposure (Warren and Matheson, 2018). For intracellular patch-clamp recordings from individual auditory neurons in the abdominal ear, Müller’s organ attached to the internal side of the tympanum was excised from the first abdominal segment by cutting around the small rim of cuticle surrounding the tympanum with a fine razor blade. The trachea and the auditory nerve (Nerve 6) were cut with fine scissors (5,200–00, Fine Science Tools), and the trachea and connective tissue were removed with fine forceps. This preparation allowed perfusion of saline to the internal side of the tympanum, necessary for water-immersion optics for visualizing Müller’s organ and the auditory neurons to be patch-clamped, and concurrent acoustic stimulation to the dry external side of the tympanum. The inside of the tympanum including Müller’s organ was constantly perfused in extracellular saline. Dissection, protease treatment, and recordings took ∼60 min for each locust ear.

To expose Group III auditory neurons for patch-clamp recordings, a solution of collagenase (0.5 mg/mL) and hyaluronidase (0.5 mg/mL) (C5138, H2126, Sigma Aldrich) in extracellular saline was applied onto the medial-dorsal border of Müller’s organ through a wide (12 μm) patch pipette to digest the capsule enclosing Müller’s organ and the Schwann cells surrounding the auditory neurons. Gentle suction was used through the same pipette to remove the softened material and expose the membrane of Group III auditory neurons. The somata were visualized with a Cerna mini microscope (SFM2, Thor Labs), equipped with an infrared LED light source and a water-immersion objective (NIR Apo, ×40, 0.8 numerical aperture, 3.5-mm working distance, Nikon) and multiple other custom modifications. For a full breakdown of the microscope components and how to construct a custom patch-clamp microscope for ∼£12 k contact Ben Warren.

### Whole-cell patch-clamp recordings

Electrodes with tip resistances between 3 and 4 MΩ were fashioned from borosilicate class (0.86 mm inner diameter, 1.5 mm outer diameter; GB150-8P, Science Products GmbH) with a vertical pipette puller (PC-100, Narishige). Recording pipettes were filled with intracellular saline containing the following (in mM): 170 K-aspartate, 4 NaCl, 2 MgCl2, 1 CaCl2, 10 HEPES, 10 EGTA, and 20 TEACl. Intracellular tetraethylammonium chloride was used to block K^+^ channels necessary for isolation of the transduction current. To further isolate and increase the transduction current, we also blocked voltage-gated sodium channels with 90 nM TTX in the extracellular saline. The addition of ATP to the intracellular saline did not alter the electrophysiology of the recordings so it was omitted. During experiments, Müller’s organs were perfused constantly with locust saline (same as that used for metabolic assays). The saline was adjusted to pH 7.2 using NaOH. The osmolality of the intracellular and extracellular salines’ were 417 and 432 mOsm, respectively.

Whole-cell voltage-clamp recordings were performed with an EPC10-USB patch-clamp amplifier (HEKA-Elektronik) controlled by the program Patchmaster (version 2 × 90.2, HEKA-Elektronik) running under Microsoft Windows (version 7). Electrophysiological data were sampled at 50 kHz. Voltage-clamp recordings were low-pass filtered at 2.9 kHz with a four-pole Bessel filter. Compensation of the offset potential was performed using the “automatic mode” of the EPC10 amplifier, and the capacitive current was compensated manually. The calculated liquid junction potential between the intracellular and extracellular solutions was also compensated (15.6 mV; calculated with Patcher’s-PowerTools plug-in from www3.mpibpc.mpg.de/groups/neher/index.php?page = software). Series resistance was compensated at 77% with a time constant of 100 µs. The resting potential was measured directly (within 10 s) after whole-cell recordings were established by changing the clamped voltage until the current was zero. We measured the amplitude of the discrete depolarizations by measuring the largest three discrete depolarizations at a −100-mV holding potential. To measure the standing current, we measured spontaneous currents for 0.5 s at a holding potential of −60 mV. We integrated the area underneath the current at the baseline (i.e., periods with no discrete depolarizations, See Figure 4B). For all patch-clamp electrophysiological analysis, we used Igor Pro 9 (Wavemetrics Inc.).

### RNA extraction

Müller’s organs from the tympanal ears of locusts were extracted as previously described in metabolic assay. Four groups of 15 male locusts ∼10 days post their last moult and four groups of 15 male locusts ∼24 days post their last moult were used (eight groups in total). Müller’s organs were snap frozen by wiping them onto a frozen pestle that rested in an Eppendorf tube submerged in liquid nitrogen. The frozen samples were then homogenized by hand with a pestle for 3 min. Then 10 μL of TRIzol was added to the samples, and homogenized for 3 min further at room temperature. Next, 490 μL of TRIzol was added to the samples and mixed gently by pipetting. Samples were then centrifuged for 10 min at 12,000 g. Supernatant of the sample was collected, and left to incubate at room temperature for 5 min. Next, 100 μL of chloroform added to the samples, and the sample was then vortexed. Samples were incubated for 3 min at room temperature, and then centrifuged for 20 min further at 12,000 g. The aqueous phase was then separated into a fresh RNase-free tube, along with 1 μL of glycogen and 250 μL of isopropanol. The samples were then mixed gently and left to incubate at room temperature. Samples were then centrifuged for 10 min at 12,000 g. The supernatant was then removed, and the RNA was then washed with 500 µL of 75% ethanol. The pellet was then dislodged via vortexing, before centrifuging again at 7,500 g for 5 min. Supernatant was again removed, and the pellet was air dried until it began to turn clear (approx. 2 min). Samples were then suspended in 10 μL of RNase-free water and incubated for 5 min at 55 °C. RNA was quantified via nanodrop, and quality control was carried out on a bioanalyser. Samples were then stored at −80°C.

### Bioinformatic analysis

Samples were sequenced and quality trimmed on the Illumina platform by Novogene sequencing (Cambridge) with 150 bp paired ends at a read depth of 60 million. Read quality was checked using FastQC (v0.11.5) (Andrews, 2010). RNA reads were aligned to the iqSchGreg1.2 reference genome (NCBI) using STAR 2-pass method (v2.7.9a) (Dobin et al., 2015), and the resultant bam files were sorted using SAMtools (v1.9) (Li et al., 2009). Gene counts were generated using HTSeq (Anders et al., 2015), and differential expression analysis was performed using DESeq2 (v1.28.1) (Love et al., 2014) on Rstudio (4.2.1), with p-adj <0.05. GO terms were generated for genes in the SchGreg1.2 using the eggNOG 5.0 web tool (Huerta Cepas et al., 2019). GO term enrichment was performed using topGO (Alexa & Rahnenfuhrer, 2022) FDR <0.05. GO term circle plots were generated using the GOPlot R package (Walter et al., 2015). Orthologous genes were determined using the OrthoFinder tool (Emms & Kelly, 2023), based on the locust iqSchGreg1.2 and *Drosophila* GCA_000001215.4_Release_6 reference genomes.

### Power analysis

We designed experiments that formed Figures 3, 4, to have a power above 95%, which gives an ability to detect a difference between young and old or control and conditioned locusts or control and experimental Müller’s organs of 95% (if these series of experiments were run an infinite amount of times). Our false negative rate, or type II error probability was <5% (1-power) (probability of not finding a difference that was there). Our false positive rate or type II error (probability of finding a difference that was not there) was determined by our *p*-values, which was set at 0.05. In order to calculate the power, we used the raw data and effect size reported by Warren et al. (2020) for hook electrode recordings of spontaneous nerve activity (Figure 3). This was also carried out for whole-cell patch-clamp recordings from individual auditory neurons of Müller’s organ (Figure 4). There exists no analytical methodology for conducting power calculations on linear mixed effect models (LMEMs). Therefore, we generated a dataset simulated from the raw data of Warren et al. (2020), fitted a linear mixed effect model, and then, ran repeated simulations of the LMEM 1000 times. We used the proportion of times that the LMEM reported a difference to calculate the power. For this paper, the experiments that measured differences in hook electrode spontaneous nerve activity and the spontaneous transduction channel openings (termed discrete depoarlizations) all had at least 95% power when using n numbers of 9 and 12, respectively. This simulated power analysis also, only applies for the effect size reported by Warren et al. (2020) and may be lower if the actual effect size, in this paper, was reduced. Models were fitted in R (Version 2.4.3), on a Windows PC running Windows 10 using the package *LME4* (Bates et al., 2015), and simulations were run with the package *simr* (Green and Macleod, 2016).

As metabolism has never been measured in auditory tissue, we had no effect size to base a power analysis for the collection of age-related changes in the metabolic rate of Müller’s organ. Instead, we decided to collect samples from a high number of locust ears (∼500) to stand the best chance of detecting a difference (Figure 2). We display Cohen’s d effect size in all datasets, where a difference was detected so that this effect size can be used for future power calculations. This same approach was used for our attempt to find a correlation between sound-evoked auditory nerve activity and metabolism, exploiting the natural variation in individual Müller’s organs (Figure 5).

### Statistical analysis

Throughout the manuscript, n refers to the number of recorded neurons and N refers to the number of Müller’s organ preparations used to achieve these recordings (i.e., n = 10, N = 6 means that 10 neurons are recorded from six Müller’s organs). All n numbers are displayed on the figures for clarity. The spread of the data is indicated by 1 standard deviation as the standard deviation indicates the spread of the data, unlike standard error. Median and Q1 and Q3 are displayed by bars when individual measurements are plotted. For all hook electrode recordings (unless otherwise stated) and 80% of patch clamp recordings the treatment of the locust (noise-exposed or control) was blinded to the experimenter; lone working conditions, due to COVID-19 restrictions, made complete blinding impossible. All data either remained blinded or were recoded to be completely blind when analyzing the data to avoid unconscious bias.

To test for differences and interactions between control, noise-exposed, and aged locusts, we used either a linear model (LM) or linear mixed effects model (LMEM), with treatment and age as fixed effects and locust identity and SPL as a random intercept, when repeated measurements are reported. Models were fitted in R (Version 3.4.3) with the package *LME4* (Bates et al., 2015). The test statistic for these analyses (t) was reported with the degrees of freedom (in subscript) and *p*-value, which were approximated using the Satterthwaite equation (lmerTest package) (Kuznetsova et al., 2017). We report Cohen’s d effect size for significant differences. Curves were fitted to the data using the *drm* package in R for patch-clamp and hook electrode recordings (Ritz et al., 2015). The *drm* package was also used to compute t and *p*-values when comparing control and noise-exposed four-part log-linear models. F statistics of the log-linear model fits were computed by excluding treatment (noise-exposed or control) as a factor. Higher F statistics denote a stronger effect of treatment.

In order to compare responses between control locusts and aged, starved or cold-reared locusts across SPLs, we adopted an approach first implemented in pharmacology research. In our work the “dose-response curves” are equivalent to SPL-auditory response curves. This allowed us to maximize the information contained in each dataset and to quantitatively compare model parameters, such as Hill coefficient (steepness of the slope), maximal asymptote (maximum *σ* ratio), and inflexion point (σ ratio at the steepest part of the slope). We did this using the *drm* function of the *drc* package (Version 3.1-1, Ritz et al., 2015).

We fitted four-part log-linear models to each individual locust’s auditory response, with auditory nerve responses (σ ratio) as the dependent variable with treatment (control or starved/cold-reared) and SPL as the independent variables. Where relevant, a fixed effect is also calculated for the locust sex, and the models for each individual locust are adjusted accordingly. The t and *p*-values are reported for each model parameter: Hill coefficient, maximal asymptote, and inflexion point are shown on each graph in Figures 6, 7. The equation of the four-parameter log-linear fits is

$$y=d+\frac{c-d}{1+{\left(\frac{x}{e}\right)}^{b}}.$$

Here, *Y* is the *σ* ratio, *b* is the slope at the inflection point, *c* is the lower asymptote, *d* is the higher asymptote, and *e* is the SPL (or *X* value) producing a response halfway between *b* and *c*.

To test whether the factor of treatment (starved or cold-reared condition or control and metabolic rate) significantly affected the auditory nerve response, we compared the aforementioned model to a model in which treatment was omitted as an independent variable, using the *anova* function (Ritz et al., 2015). This gave an F statistic labeled on each graph in Figures 6, 7. Cohen’s d was then used to calculate the effect size difference at the most different dB SPL. This dB SPL was then used in Figure 5C.

## Data Availability

The datasets presented in this study can be found in online repositories. The names of the repository/repositories and accession number(s) can be found at: Mendelely DOI: 10.17632/jgmwdvx9f7.1. Transcriptomic data can be accessed through NCBI: Eight Accession numbers: SRX19218695-SRX19218702.
